# CalpB modulates border cell migration in *Drosophila* egg chambers

**DOI:** 10.1186/1471-213X-12-20

**Published:** 2012-07-24

**Authors:** Endre Kókai, Ferencz Sándor Páldy, Kálmán Somogyi, Anil Chougule, Margit Pál, Éva Kerekes, Péter Deák, Péter Friedrich, Viktor Dombrádi

**Affiliations:** 1 Department of Medical Chemistry, Faculty of Medicine, University of Debrecen, Nagyerdei krt. 98, Debrecen H-4032, Hungary; 2 Institute of Genetics, Biological Research Center, Hungarian Academy of Sciences, Szeged, Hungary; 3 Institute of Biochemistry, Biological Research Center, Hungarian Academy of Sciences, Szeged, Hungary; 4 Institute of Enzymology, Hungarian Academy of Sciences, Budapest, Hungary

**Keywords:** Cell motility, Proteolysis, Focal adhesion, Calpain, Talin, Integrins

## Abstract

**Background:**

Calpains are calcium regulated intracellular cysteine proteases implicated in a variety of physiological functions and pathological conditions. The *Drosophila melanogaster* genome contains only two genes, *CalpA* and *CalpB* coding for canonical, active calpain enzymes. The movement of the border cells in *Drosophila* egg chambers is a well characterized model of the eukaryotic cell migration. Using this genetically pliable model we can investigate the physiological role of calpains in cell motility.

**Results:**

We demonstrate at the whole organism level that *CalpB* is implicated in cell migration, while the structurally related *CalpA* paralog can not fulfill the same function. The downregulation of the *CalpB* gene by mutations or RNA interference results in a delayed migration of the border cells in *Drosophila* egg chambers. This phenotype is significantly enhanced when the focal adhesion complex genes encoding for α-PS2 integrin ( *if*), β-PS integrin ( *mys*) and talin ( *rhea*) are silenced. The reduction of *CalpB* activity diminishes the release of integrins from the rear end of the border cells. The delayed migration and the reduced integrin release phenotypes can be suppressed by expressing wild-type talin-head in the border cells but not talin-head^R367A^, a mutant form which is not able to bind β-PS integrin. CalpB can cleave talin *in vitro,* and the two proteins coimmunoprecipitate from *Drosophila* extracts.

**Conclusions:**

The physiological function of *CalpB* in border cell motility has been demonstrated in *vivo*. The genetic interaction between the *CalpB* and the *if*, *mys,* as well as *rhea* genes, the involvement of active talin head-domains in the process, and the fact that CalpB and talin interact with each other collectively suggest that the limited proteolytic cleavage of talin is one of the possible mechanisms through which CalpB regulates cell migration.

## Background

Calpains are intracellular cysteine proteases that are tightly regulated both temporally and spatially, as they require the binding of Ca^2+^ for their proteolytic function, and they are activated in a highly localized manner within the cells. Calpains are widespread and are implicated in a variety of physiological functions (signal transduction, cell motility, cell proliferation and differentiation, apoptosis, cell growth, cytoskeletal remodeling, membrane fusion) [1]. They are also associated with a diverse range of pathological conditions such as Alzheimer’s, Huntington’s and Parkinson’s diseases, multiple sclerosis, ischemic and traumatic brain injury, cancer, muscular dystrophy, cataracts, stroke and diabetes [2,3]. In mammals, they form a superfamily which is comprised of 15 cysteine protease catalytic subunits (including the homologue of *Drosophila* SOL), 2 regulatory subunits and the inhibitor, calpastatin. Some of the calpains are expressed ubiquitously, while others have a restricted tissue distribution [4].

The role of calpains in the migration of Chinese Hamster Ovary (CHO) cells was demonstrated with the aid of cell-permeable calpain inhibitors and a cell line expressing reduced level of Capn1 [5]. Both conditions leading to reduced calpain activity blocked cell migration at higher fibrinogen concentrations, inhibited the cell-substratum detachment at the trailing edge of the cells, and decreased the release of integrins from the cell membrane. From this data it was concluded, that calpain destabilizes the connection between the adhesion complexes and the cytoskeleton at the rear of the moving cells. Palecek et al. confirmed that a calpain inhibitor decreased integrin release behind the cells when the rear detachment was the rate limiting step of migration [6]. The downregulation of a mammalian Capn2 by RNA interference lead to similar results: the dissociation rate of adhesion complexes was decreased [7]. Moreover, when a calpain-resistant Talin1 was expressed in cells with Talin1 null background, the rate of disassembly of the focal adhesion complexes was also lower indicating that adhesion turnover was regulated by the Capn2-mediated cleavage of Talin1 [7]. In addition to talin calpain has a whole array of motility related substrates [4]. For example in the focal adhesion complexes two essential scaffolding molecules, focal adhesion kinase FAK [8] and paxillin [9] have been recently identified as physiological targets of calpain cleavage. Despite remarkable progress, our knowledge about the role of individual calpains during cell migration is incomplete due to the large number and variety of calpains as well as their potential substrate proteins.

In contrast to mammals, the *Drosophila* genome contains only four genes encoding calpain-related proteins. Out of these, only *CalpA* and *CalpB* encode canonical, active calpain enzymes. The *CalpC* gene product is an inactive protease, while *CalpD (sol)* encodes an atypical calpain molecule [10]. The structural characteristics of the *Drosophila* calpains are very similar to that of the mammalian enzymes, although they have two distinguishing features: the CalpA protein carries a 76 amino acid long hydrophobic insert in its C-terminal end, and the CalpB protein has an unusually long N-terminal tail of 224 amino acids. The exact molecular functions of these extra sequences are not known. There are two other distinctive enzymatic characteristics of the *Drosophila* calpains: they do not need small regulatory subunits for their catalytic activity, and their proteolytic function is not regulated by an intrinsic inhibitor as the *Drosophila* genome contains neither regulatory subunit nor calpastatin orthologs. In addition to the smaller number of calpain genes, *Drosophila* also offers a genetically well-tractable model system in which the functions of calpains and their molecular interactions can be studied at the organism level.

Movement of the border cells in *Drosophila* egg chambers is a well characterized genetic model of the eukaryotic cell migration [11,12]. Using this model, here we demonstrate that *CalpB* but not the structurally related *CalpA* has an important modulator function during *in vivo* cell migration. Based on our molecular-genetic and biochemical results we suggest that one of the potential targets of the CalpB catalyzed limited proteolysis is talin, an important component of the focal adhesion complex.

## Results

### CalpA and CalpB are not essential genes in Drosophila melanogaster

**Figure 1:**
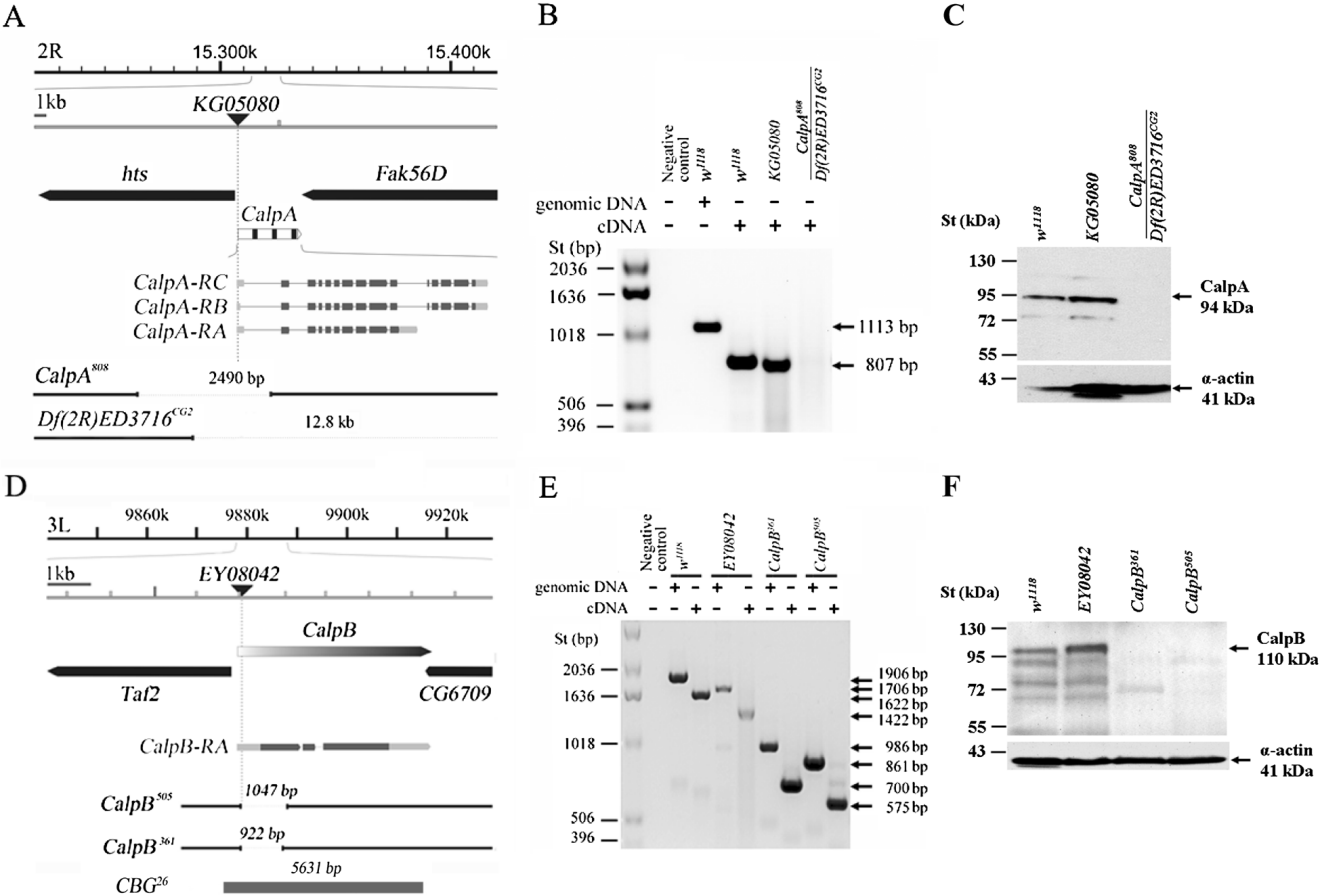
**Isolation of*CalpA*and*CalpB*alleles. (A**) Genomic organization of the *CalpA* locus at cytological position 56D5. The position of the *CalpA*^*KG05080*^ P-element, inserted 948 bp upstream from the *CalpA* translation initiation site, is indicated. The breakpoints and the sizes of the *CalpA*^*808*^ and the *Df(2R)ED3716*^*CG2*^ deficiencies are shown at the bottom. The boxes represent exons in the gene and in the primary transcripts. (**B**) RT-PCR detects *CalpA* mRNA in the *w*^*1118*^ control strain and in the *P{SUPor-P}CalpA*^*KG05080*^ insertion line but not in the *CalpA*^*808*^/*Df(2R)ED3716*^*CG2*^ transheterozygotes. Arrows indicate wild-type genomic and complementary DNA RT-PCR fragments. (**C**) Western blotting of adult whole fly extracts reveals CalpA protein in the *w*^*1118*^ control strain and in the *P{SUPor-P}CalpA*^*KG05080*^ insertion line but not in the *CalpA*^*808*^/*Df(2R)ED3716*^*CG2*^ transheterozygotes. The lower panel shows loading controls; the arrows mark the positions of CalpA and α-actin bands. **(D**) Genomic organization of the *CalpB* locus at cytological position 67D1. The location of the *CalpB*^*EY08042*^ P-element, inserted 175 bp upstream from *CalpB* ATG, is indicated. The breakpoints and the sizes of the *CalpB*^*505*^ and *CalpB*^*361*^ deficiencies are shown at the bottom together with the size of the *CBG*^*26*^ genomic DNA fragment used for the rescue of the mutant phenotypes. The boxes indicate the exons in the primary transcript. (**E**) RT-PCR reveals the presence of truncated *CalpB* mRNA in the *CalpB*^*505*^ and *CalpB*^*361*^ homozygotes. The calculated sizes of the PCR products are designated by the arrows on the right. (**F**) No CalpB-specific signals can be detected in the extracts from adult *CalpB*^*505*^ homozygous mutants by Western blotting. The arrows denote the positions of the full length 110 kDa CalpB in the upper panel, and the α-actin loading control bands in the lower panel, respectively.

The *Drosophila melanogaster* genome contains two catalytically active canonical calpain genes: *CalpA* and *CalpB*, but little is known about their *in vivo* roles. To characterize their functions, we generated deletions disrupting each gene. For the *CalpA*, the *P{SUPor-P}CalpA*^*KG05080*^ transposon, inserted 948 bp upstream from the *CalpA* translation initiation site, was mobilized to obtain imprecise excisions. A single line, *CalpA*^*808*^*,* carrying a 2490 bp deletion, was identified by PCR. Mapping the breakpoints by sequence analysis revealed that the deficiency removed not only the P element, but 459 bp from the *CalpA* gene including the first untranslated exon and a 1907 bp sequence from the adjacent *hts* gene (Figure 1A). In *CalpA*^*808*^ homozygous mutant flies, the *CalpA mRNA* and the CalpA protein were not detectable by RT-PCR and Western blot, respectively (data no shown). Homozygous *CalpA*^*808*^ flies are viable, displaying no external morphological phenotypes, but the females are completely sterile; they do not lay eggs. In their ovaries, egg chamber development is arrested at early stages, reminiscent of the egg chamber phenotype of the female sterile mutants of the hts gene (data not shown, [13]). This female sterility was complemented in the *CalpA*^*808*^/*Df(2R)ED3716*^*CG2*^ transheterozygotes. The 12.8 kb long Df(2R)ED3716^*CG2*^ deficiency completely eliminates the *Fak56D* and the *CalpA* genes and 834 bp from the *hts* gene (Figure 1A) [14] . Both the *CalpA*^*808*^ and the *Df(2R)ED3716*^*CG2*^ deficiencies affect the regulatory sequences of the *hts*, which in *CalpA*^*808*^ results in female sterility. Presumably, *Df(2R)ED3716*^*CG2*^ can complement this phenotype because it removes a shorter segment of hts and it does not eliminate those regulatory sequences that are responsible for the female sterility. From these data, we concluded that the female sterility of *CalpA*^*808*^ originates from the *hts* gene. However, in a more detailed analysis, it appeared that in *CalpA*^*808*^/ *Df(2R)ED3716*^*CG2*^ transheterozygous ovaries 10% of the egg chambers display a mild dumpless phenotype (data not shown) indicating that the *Df(2R)ED3716*^*CG2*^ deficiency can only partially complement the female sterility in *CalpA*^*808*^. In the *CalpA*^*808*^/*Df(2R)ED3716*^*CG2*^ transheterozygotes, similarly to the *CalpA*^*808*^ homozygotes, no *mRNA* and no CalpA protein were present (Figure 1B and C). Since *CalpA*^*808*^/*Df(2R)ED3716*^*CG2*^ flies are viable, *CalpA* can be considered a non-essential gene.

To generate mutations in the *CalpB* gene, the *P{EPgy2}CalpB*^*EY08042*^ P-element, inserted 175 bp upstream from *CalpB* ATG, was mobilized. Several imprecise excision lines were recovered, and the breakpoints of two alleles ( *CalpB*^*361*^ and *CalpB*^*505*^) were identified by sequencing: both of them disrupted the *CalpB* gene only (Figure 1D). The longer deficiency, *CalpB*^*505*^*,* removes the P-element and 1047 bp from the *CalpB* gene. As a result of this deletion, the *CalpB*^*505*^ allele lacks the first 873 bp of the coding sequences of the *CalpB* gene. *CalpB*^*361*^ encompasses a shorter, 922 bp deletion with an identical starting point. In both the *CalpB*^*361*^ and the *CalpB*^*505*^ homozygous flies, truncated mRNAs were detected by RT-PCR (Figure 1E), but no full length or truncated CalpB protein bands were discernible in *CalpB*^*505*^ by immunoblots (Figure 1F). The *CalpB*^*505*^ deficiency, together with the other isolated deletion alleles (including *CalpB*^*361*^), is homozygous viable and fertile, displaying no visible external abnormalities indicating that *CalpB*, similarly to *CalpA*, is not an essential gene in *Drosophila*. To test the possible functional redundancy of the *CalpA* and the *CalpB* genes, a double mutant line ( *CalpA*^*808*^/*Df(2R)ED3716*; *CalpB*^*505*^*/CalpB*^*505*^) was generated which was also fully viable and fertile demonstrating that the *CalpA* and *CalpB* genes do not fulfill any common essential functions.

### CalpB but not CalpA modulates border cell migration in egg chambers

**Figure 2:**
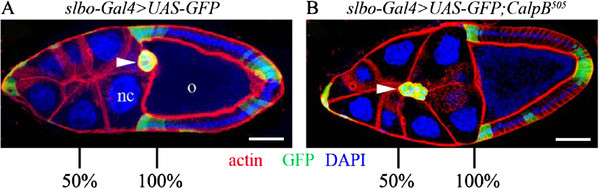
***CalpB*mutation delays border cell migration.** Confocal micrographs of ( **A**) control ( *w*^*1118*^) and (**B**) *CalpB*^*505*^*/CalpB*^*505*^ egg chambers at stage 10A are triple labeled with phalloidin-TRITC (red) for the actin network, with GFP (green) to show the slbo-Gal4 expression patterns and with DAPI (blue) for nuclei. The nurse cells (nc) and the oocyte (o) are indicated in panel A. The arrowheads show the positions of the border cell clusters, and the bars represent 30 μm The percentage scale represent the relative distances on the migration path between the anterior ends of the egg chambers and the oocytes. Note that in the wild-type egg chamber, the border cell cluster is located dorsally.

**Figure 3:**
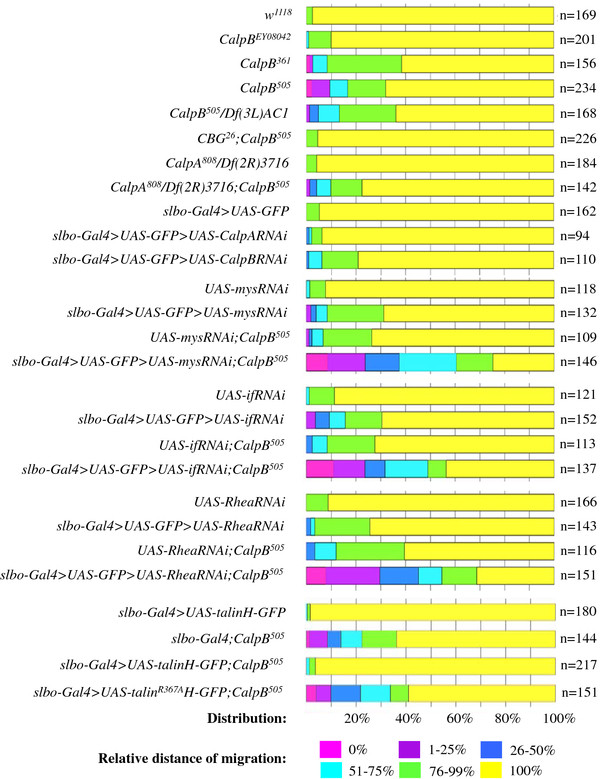
**Quantification of the border cell migration defects.** The alterations of border cells movements caused by *CalpB* and *CalpA* mutations, by RNA interference, or by the overexpression of talin heads are analyzed as in Figure 2. Colored bars indicate the proportion of stage 10A egg chambers in which border cells migrate 0% (pink), 1-25% (purple), 26-50% (dark blue), 51-75% (light blue), 76-99% (green) and 100% (yellow) of the wild-type distance. Thus, the color scale shows the relative distance of posterior border cell movement. Note that in the *w*^*1118*^ control the majority of border cells travel the 100% distance to reach the oocyte by stage 10A, and the next category (76-99% range) represents only a relatively mild migration defect. The genotypes and the corresponding numbers of the egg chambers examined are displayed at the left and the right of the chart, respectively.

Since *CalpB* is not required for vital functions, we investigated whether the *CalpB* mutants display any other developmental perturbations. Previous studies revealed that the follicular cells of the *Drosophila* egg chambers are particularly enriched in CalpB protein, and the gene is also highly expressed in the migrating border cells [15], which originate from the follicular cells. Guided by this cellular localization and by the literature showing that calpains are involved in the regulation of mammalian cell motility, we examined whether the egg chambers from flies carrying *CalpB* mutations display any abnormalities in the migration of border cells. In wild-type egg chambers, the border cells usually reach the anterior end of the oocyte by stage 10, while in *CalpB* homozygous mutants a significant portion of the border cells (32-38%) do not complete their migration by this stage (Figure 2), indicating that the lack of *CalpB* delays but does not arrest border cell migration. The quantitative analysis of the data (Figure 3) revealed no significant difference between the *P{EPgy2}CalpB*^*EY08042*^ P-element insertion line and the wild type. The extent of the delay in border cell migration was similar in the *CalpB*^*505*^ and the *CalpB*^*361*^ lines. In these mutants, the border cells migrated in the correct direction; no guidance defect was observed. In a *CalpB*^*505*^ hemizygote containing a large chromosomal deficiency (*Df(3 L)AC1*) that removes the entire *CalpB* gene, the border cell migration was also delayed, just as much as in the homozygotes. To verify that the disruption of the *CalpB* gene is responsible for the slow migration, the genomic rescue fragment of the *CalpB* gene, termed *CBG*^*26*^, was introduced into the *CalpB*^*505*^ mutant. The rescue fragment restored the wild-type phenotype proving that the lack of *CalpB* gene is indeed responsible for the less efficient border cell movement.

As *CalpB* mutations do not block border cell migration completely, the possibility emerged that the other calpain gene, i.e. *CalpA,* may also be involved in the process. To test this hypothesis, egg chambers from *CalpA*^*808*^/*Df(2R)ED3716*^*CG2*^ transheterozygous flies were investigated. Although CalpA protein is not expressed in these flies, the border cells migrate normally (Figure 3). Furthermore, the *CalpA* and *CalpB* double mutants display a phenotype characteristic of *CalpB* single mutant indicating that *CalpA* is not required for border cell migration. We confirmed these results by downregulating the expression of the *CalpA* and *CalpB* genes in the border cells via RNA interference. The *slbo-Gal4* driver was used to induce *CalpA* or *CalpB* specific *RNA* interference. *CalpA RNAi* did not influence border cell migration, but *CalpB RNAi* resulted in a similar, delayed migration phenotype as observed in the *CalpB* mutant (Figure 3). Consistently with earlier reports [16,17] the *slbo-Gal4* driver operated rather specifically, it induced GFP expression exclusively in border cells, posterior polar cells, and some follicular cells at the anterior side of the oocytes (Figure 2). Consequently, the *RNA* interference results not only confirm the mutant phenotype, but also reveal that *CalpB* is required only in the border cells (and not in nurse cells) in order to regulate their migration.

### CalpB interacts with the focal adhesion complex proteins

To investigate if the reduced cell motility was related to the focal adhesion complex proteins we silenced the expression of the genes encoding *Drosophila* integrins and talin (Figure 3). As expected from the published data [17] the border cell specific silencing of the *myospheroid* ( *mys*, β-PS integrin) gene by RNA interference slowed down migration. Similar results were obtained when the *inflated* ( *if,* α-PS2 integrin) and the *rhea* (talin) genes were silenced: in all cases approx. 30% of the border cells were delayed. In the same RNA interference lines, border cell migration was normal when the *slbo-Gal4* driver was absent indicating that the slower migration is due to the reduced expression of the affected genes. After establishing the phenotypes of the silenced lines we tested the genetic interrelationship between the focal adhesion protein coding genes and *CalpB*. The frequency and severity of the migration defect increased when the *mysif* and *rhea* genes were downregulated in a homozygous *CalpB*^*505*^ background (Figure 3). RNA interference flies without the *slbo-Gal4* driver in the *CalpB*^*505*^ background showed the expected 30% mutant migration phenotype resulting from the deletion of the *CalpB* gene alone. Thus, our data provide genetic evidence for the functional interaction of *CalpB* with the *mysif*, and *rhea* genes.

It is known, that the migration of the border cells takes place in two phases: first they proceed posteriorly from the anterior end of the egg chamber, then, after reaching the oocyte, they move towards the dorsal side, and by stage 10B they are positioned next to the germinal vesicle. The analysis of the second stage of migration is of interest, since some mutations, like *gurken* influence specifically the dorsal movement of the border cells with little effect on the initial posterior migration [18]. By investigating the egg chambers at stage 10B, we found that a significant portion of the border cells (46%) in the CalpB^*505*^ mutants displayed a delay in the dorsal movement (Additional file 1). The dorsal migration of the border cells was even more severely delayed when the *mysif*, and *rhea* genes were silenced by RNA interference in the *CalpB*^*505*^ mutants: at stage 10B most of the cells (92.0%, 73.7%, and 83.6%, respectively) remained at the midline of the egg chambers. Our data collectively indicate that the *CalpB* gene is involved both in the posterior and in the dorsal migration of the border cells and interacts with the cell adhesion genes *mysif*, and *rhea*.

**Figure 4:**
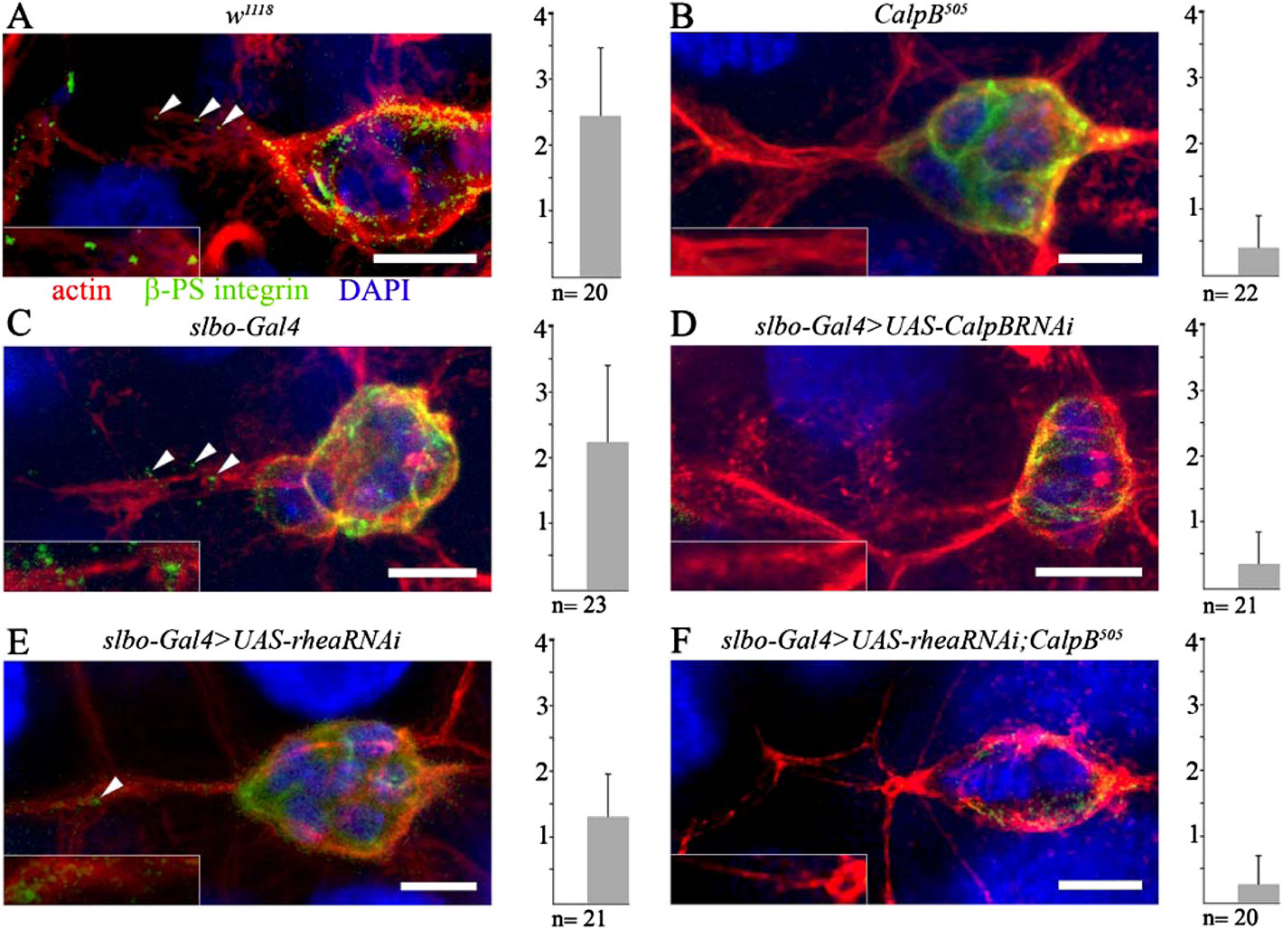
**Quantification of integrins released from the border cells during migration.** In the confocal micrographs of ( **A**) wild-type ( *w*^*1118*^), (**B**) *CalpB*^*505*^, (**C**) *slbo-Gal4*, ( **D**) *slbo-Gal4 >UAS-CalpBRNAi*, ( **E**) *slbo-Gal4 >UAS-rheaRNAi*, ( **F**) *slbo-Gal4 >UAS-rheaRNAi*; *CalpB*^*505*^ egg chambers, the areas of egg chambers around and behind the border cells are labeled with phalloidin-TRITC (red) for actin, with anti-β-PS integrin (green), and with DAPI (blue) for nuclei. In the inserts, the area directly behind the border cells is magnified. The movement of the border cells proceeds from the left to the right, and the released integrins left behind the cells appear as discrete spots (labeled by arrowheads) at the left sides of the images. Representative pictures of 20–23 independent experiments are shown. The calculated average numbers of integrin spots at the rear of the border cells per egg chamber, and the corresponding number of investigated egg chambers (n) are indicated by the diagrams at the right side of each panel. Note that only the spots falling into the cells’ pathway were scored, and groups of closely situated spots were counted as a single “spot”. Bars indicate 100 μm.

Since in cultured cells the reduction of calpain activity not only delayed motility but also reduced the amount of integrins released from the trailing cell membrane [6] we analyzed the role of *CalpB* in this process under *in vivo* conditions (Figure 4). The egg chambers were stained for β-PS integrin and the released integrins were visualized as discrete spots behind the border cells in the anterior part of the wild-type egg chambers (Figure 4A). In the *CalpB*^*505*^ mutant, the number of the β-PS integrin-containing spots was significantly reduced (Figure 4B). The downregulation of the *CalpB* gene by RNA interference had the same effect as the *CalpB*^*505*^ deletion: in the control *slbo-Gal4* driver strain the number of the released β-PS integrin spots was similar to that of the wild type (Figure 4C) while in the *slbo-Gal4 >CalpB RNAi* silenced strain this number was close to that of the *CalpB*^*505*^ mutant (Figure 4D). Thus, *CalpB* modulates integrin function *in vivo* in the border cells during their migration. As we found that the downregulation of the talin-encoding gene, *rhea,* also had a delaying effect on border cell migration, and this effect was aggravated in a *CalpB*^*505*^ background (Figure 3), we investigated the effects of the same conditions on integrin release. The silencing of *rhea* alone in the border cells decreased integrin release to about 60% of the wild-type level (Figure 4E) while, the integrin spots were hardly detectable when *rhea* was silenced in the *CalpB*^*505*^ mutant background (Figure 4F). Our data lend additional support for the interaction between *rhea* and *CalpB*, but at the same time indicate that CalpB must act *via* additional targets, since the talin silencing phenotype (Figure 4E) is significantly weaker than the calpain knockdown or deletion phenotypes (Figure 4D and 4B).

### Functionally competent talin head-domain abrogates the effects of CalpB mutation

**Figure 5:**
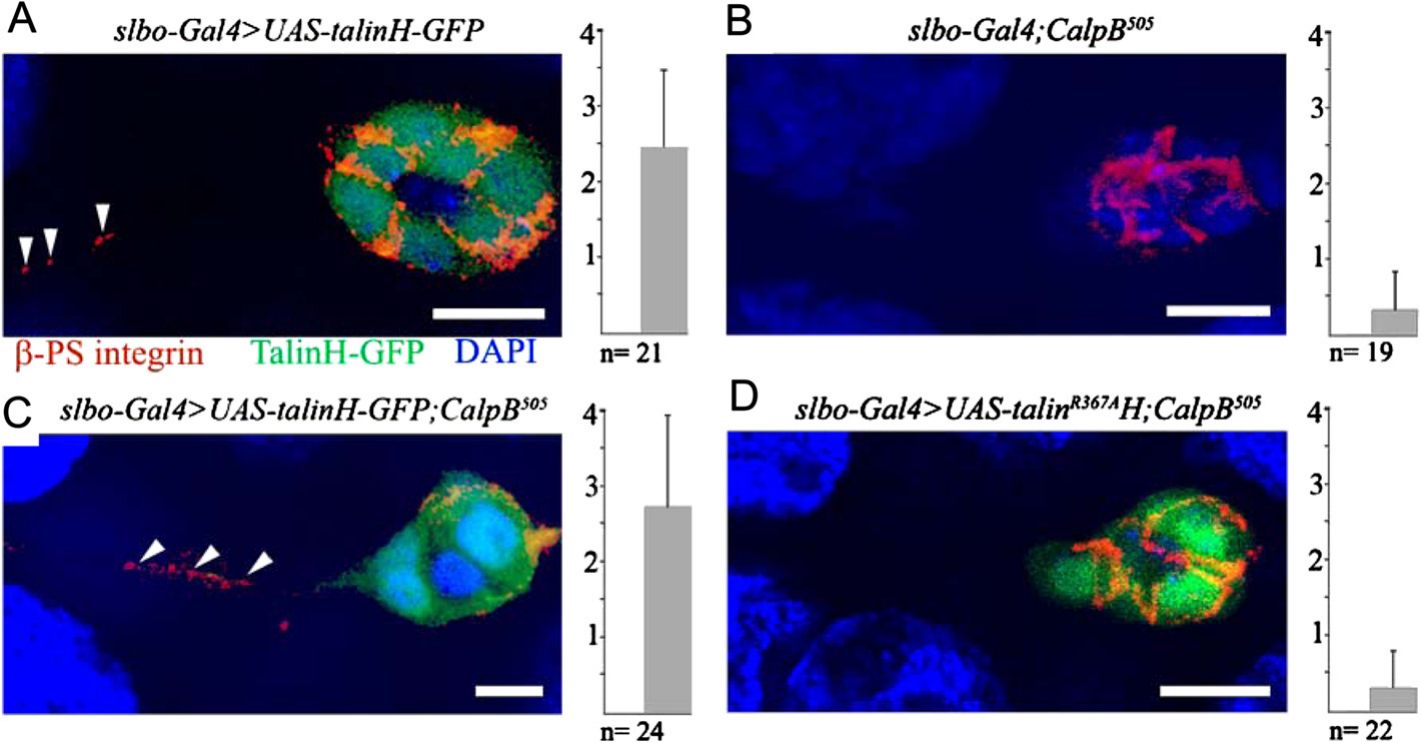
**Effect of talin head domain overexpression on integrin release from border cells.** In the confocal images of ( **A**) *slbo-Gal4 >UAS-TalinH-GFP*, ( **B**) *slbo-Gal4;CalpB*^*505*^, (**C**) *slbo-Gal4 >UAS-TalinH-GFP; CalpB*^*505*^ and (**D**) *slbo-Gal4 >UAS-TalinH*^*R367A*^*-GFP; CalpB*^*505*^ egg chambers, integrins were stained with Alexa-546 conjugate (red), TalinH-GFP fusion proteins were visualized in green and the nuclei were labeled with DAPI (blue). The images were analyzed as described in Figure 4. Bars represent 100 μm.

To dissect the nature of the *CalpB-rhea* interaction we utilized a construct that overexpresses the globular head domain of talin, which can bind to and activate integrins. In a wild-type genetic background, the expression of a *Talin head-GFP* fusion polypeptide in the border cells did not significantly alter the migration of the border cells, but restored the normal migration rate in *CalpB*^*505*^ mutant egg chambers (Figure 3). In addition, the expression of a mutant (*R367A*) *Talin head-GFP*[19], which is unable to bind β-PS integrin (and activate the integrin dimer), had no effect on the migration delay of the *CalpB*^*505*^ border cells (Figure 3). In correlation with the above results, we found that the amount of integrins released at the rear of the border cells was normal when the wild-type *Talin head-GFP* was expressed in the border cells (Figure 5A), in *CalpB*^*505*^ mutant border cells *Talin head-GFP* expression elevated integrin release to the wild-type level (Figure 5B vs. 5C), while the *Talin head*^*R367A*^*-GFP* expression had no effect on the *CalpB*^*505*^ mutant phenotype (Figure 5D). With these experiments, we demonstrated that overexpression of a functionally competent Talin head suppresses the characteristic phenotypes of the *CalpB* mutant border cells, suggesting that CalpB mediates cell migration *via* the proteolytic release of the head region from talin.

### Calpain directly interacts with talin

**Figure 6:**
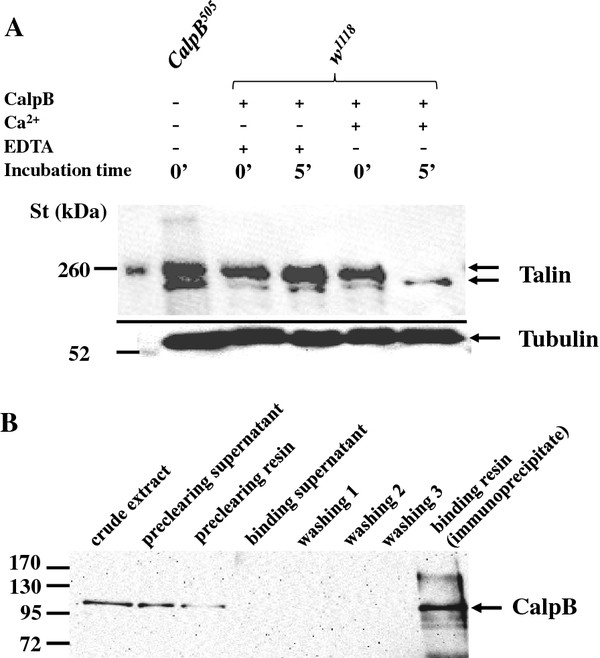
**Physical interaction between CalpB and talin in*Drosophila*.** ( **A**) Talin is an *in vitro* substrate of CalpB. Crude protein extracts of stage 2 embryos were analyzed directly (0’) or were treated with recombinant CalpB for 5 minutes (5’) in the absence and presence of 20 mM Ca^2+^ or 10 mM EDTA. An untreated extract from *CalpB*^*505*^ embryos was also tested for comparison. The proteolytic fragmentation of talin was investigated by Western blotting using a talin-specific antibody (upper section). The loading of the lanes was tested by a tubulin-specific antibody (lower section). St denotes molecular mass standards; the positions of two pre-stained bands are labeled at the left side, and arrows indicate the main immunoreactive talin bands at the right side. (**B**) Coimmunoprecipitation of talin with CalpB. Immunoprecipitations were performed in embryonic extracts with anti-Talin antibody, and the immunoblots were stained with anti-CalpB antibody. The positions of the full-length CalpB bands are marked by the arrow.

To verify that *Drosophila* talin can act as an *in vitro* substrate of CalpB, embryonic extracts were incubated with purified recombinant CalpB. In the presence of Ca^2+^, the cleavage of the full length talin was complete within 5 minutes, whereas in the presence of the Ca^2+^ chelator (EDTA) the full length talin band remained intact (Figure 6A). It should be noted, that the recombinant protease required rather high Ca^2+^ concentration for activation, but in the *in vitro* tests of calpain activity it is commonplace to use the metal ion in the non-physiological mM range [20]. The physical interaction between talin and CalpB was shown by co-immunoprecipitation (Figure 6B). After the incubation of embryonic extracts with anti-talin antibody, CalpB was detected in the precipitate. As talin is a potential substrate of CalpB, and the two proteins make a physical contact with each other, it is feasible to assume that CalpB may function *via* talin scission in the modulation of cell migration. However, we also noted that talin was partially degraded in an untreated *CalpB*^*505*^ embryo extract (Figure 6A), clearly showing that in the absence of CalpB other proteases can digest talin, even if Ca^2+^ was not added.

## Discussion

### Comparison of calpain functions in mammals and Drosophila

The analysis of *CalpA*^*808*^/*Df(2R)ED3716*^*CG2*^ hemizygotes and *CalpB*^*505*^ homozygous mutants clearly shows that both *CalpA* and *CalpB* are dispensable for viability and fertility while the features of the *CalpA* and *CalpB* double mutant prove that they do not share vital functions during *Drosophila* development. These findings were unexpected since earlier experiments with the mammalian *Capn4* gene (encoding a small calpain regulatory subunit) suggested that calpains are required for normal development in mice [21,22]. Since the lack of *Capn4* can affect both the *Capn1* and *Capn2* activities, these proteases were investigated separately by knocking out their corresponding genes. The elimination of *Capn2* resulted in early embryonic lethality [23], while the disruption of *Capn1* did not affect viability [24] indicating that from the two ubiquitous calpain genes only one (*Capn2*) is indispensable. Although the loss-of-function mutation of the *Capn1* gene did not influence viability and fertility, it affected platelet aggregation and clot retraction [24]. Similarly, disruption of the *Capn3* gene has no lethal consequences; the life span of the mutant was normal, but a moderate loss of sarcolemmal integrity could be detected in the KO mice [25]. Thus, the mammalian *Capn1* and *Capn3* genes are not essential, but play important modulator roles during development. Similarly, we observed that the *CalpB* gene in *Drosophila* is not required for essential physiological functions, but has a modifying role in border cell migration.

Although the CalpA protein is expressed in all stages of *Drosophila* development (Additional file 2) the *CalpA* gene is also dispensable for viability and fertility. It should be noted that our results do not confirm the conclusions of a recent paper [26] reporting that the downregulation of *CalpA* by the microinjection of double stranded RNAs resulted in the dorsalization of embryos: a significant portion of the larvae did not hatch and bore cuticule defects. In our case, the larvae of *CalpA*^*808*^/*Df(2R)ED3716*^*CG2*^ transheterozygotes displayed no visible dorsoventral patterning abnormalities and exhibited no cuticular defects (data not shown). They hatched, although the hatching rate of the embryos was somewhat lower than the theoretical ratio owing to dumpless eggs (10%) which did not hatch. Fontenele et al. [26] also reported that the downregulation of *CalpB* by RNA interference has similar dorsalization effects with very low hatching rate and cuticle defects. However, the embryonic development of our *CalpB* mutants was normal without any reduction in hatching (data not shown). The discrepancy may be related to the utilization of different genetic tools; we suppose that the developmental defects [26] may have been generated by some off-target effects of the microinjection based silencing approach. Since both *CalpA*^*808*^ and *CalpB*^*505*^ are protein null alleles, it is likely that neither *CalpA* nor *CalpB* are involved in the establishment of the dorsoventral axis in *Drosophila*. Nevertheless, the *Drosophila* calpains may have modulator roles in other cellular processes like cell migration, neuronal functions or phagocytosis. Indeed it has been shown recently that the downregulation of *CalpA* by RNA interference significantly decreases the S2 cell phagocytosis of *C. albicans*[27], and that *CalpB* can modify the effect of the human leukemogenic fusion protein AML1-ETO when expressed in *Drosophila* blood cells [28].

### In vivo evidence for the involvement of CalpB in cell migration

Here we provide the first *in vivo* evidence showing that calpains modulate cell motility at the whole organism level. In *CalpB* mutant egg chambers, border cells display a moderate delay during migration. In our study, we were also able to detect released β-PS integrin behind the border cells in wild-type egg chambers. In the absence of CalpB, the released β-PS integrin was markedly reduced in the egg chambers that displayed delayed migration. These results are in correlation with previous data obtained with a mammalian cell line showing that the inhibition of calpain activity decreased the rear detachment rate and gave rise to less integrins to be released from the rear of migrating cells [6]. We also noticed that the effects of *CalpB* insufficiency on cell motility were synergistically enhanced when the expression of integrins and talin, the components of the focal adhesion complex, were silenced. However, our data are at variance with an earlier paper [29] reporting that the knocking down of talin has no effect on border cell migration. This discrepancy is especially disturbing since our RNA interference construct originates from the same source (6831R-2, NIG-FLY). The only difference between the two experimental conditions was that we performed the silencing at 30°C while Llense and Martin-Blanco [29] did it at 25°C. To test the importance of the cultivation conditions, we carried out the same RNA interference experiments at both temperatures. At 30°C, the penetrance was 29%, while in parallel experiments carried out at 25°C it was only 18% (Additional file 3). We conclude that the phenotype exists at both temperatures but can be more readily detected and characterized at 30°C. Since Llense and Martin-Blanco [29] used a less graded scoring scale than we, it is possible, that they counted the small migration defect as normal. Indeed, the migration phenotypes of the single *rhea* and *CalpB* knockdown strains are mild but the combination of them results in a strong effect that reveals the role of both genes in border cell migration. Thus we are convinced that the genetic interaction between *CalpB* and the focal adhesion protein coding *mysif* and *rhea* is a solid fact that we substantiated by two independent methods. We also fund that the above interaction is important for both the posterior and dorsal components of the border cell migration, thus it affects a general aspect of cell motility.

It is more difficult to ascertain the molecular mechanisms underlying the above interactions, because the proteins that we manipulated by molecular genetic methods are members of a large complex characterized by a complicated network of direct and indirect, molecular and functional, interactions. Although we demonstrated that *Drosophila* talin is an *in vitro* substrate of CalpB, and the two proteins are in physical contact with each other in embryo extracts we do not have direct evidence for a talin cleavage mediated calpain action in the border cells. We cannot exclude the possibility that the protease acts in talin independent pathways as well, for example *via* the limited proteolysis of FAK [8], paxillin [9] or other [4] potential substrates that affect cell motility. In fact, our integrin release tests show that the effect of talin silencing is less pronounced than the effects of *CalpB* silencing or deletion (Figure 4) suggesting that besides talin other calpain targets must be involved in the process. Furthermore, we found that in the *CalpB*^*505*^ null mutant flies other unidentified proteases can also recognize talin as a substrate (Figure 6A). Disregarding the uncertainty of the exact mechanisms our finding that the overexpression of a *Talin head-GFP* construct can suppress both the delayed migration and the reduced release of integrins at the rear in *CalpB*^*505*^ border cells is intriguing, especially because only the functionally competent talin head domain was effective, the mutant variant that is unable to bind integrins had no effect. Thus calpain B action and the activation of integrins by the active talin head domains are functionally related events. Based on circumstantial evidence and analogies with the cell line experiments [7] it is tempting to speculate that CalpB generates talin heads that activate integrin clustering, and at the same time CalpB weakens the connection between integrin and the extracellular matrix at the trailing end of the cells by cleaving additional protein substrates [6]. In these ways calpain B may contribute to the tension generation and the ripping off of the integrins at the rear of the border cells and acts as a regulator of cell migration in the *Drosophila* ovaries.

## Conclusions

We demonstrated at the organism level that the canonical calpains in *DrosophilaCalpA* and *CalpB*, are dispensable for vital functions, but *CalpB* has a modulator role in the regulation of cell migration: it fine-tunes the adhesion complexes in the border cells during their migration. It is interesting to note that among the 18 mammalian calpain superfamily members, only *Capn1* and *Capn2* are involved in the regulation of cell motility [30], but only Capn2 can cleave talin *in vivo*. In an analogous way, only one of the two canonical *Drosophila* calpain genes, *CalpB,* is implicated in border cell migration, and may act *via* the digestion of talin. Thus one of the important calpain functions was most probably conserved between mammals and Drosophila.

Cell motility is an essential factor governing development [31]. The uncovering of the role of *CalpB* and the functionally competent talin head in border cell migration supports existing models and helps the better understanding of cell motility in general. Besides cell adhesion, cell spreading and cell protrusion formation are important developmental processes in which the role of *CalpB* remains to be elucidated.

## Methods

### Fly stocks

Unless indicated otherwise, all *Drosophila* stocks were maintained and crossed at 25°C according to standard procedures. Since the efficiency of the genes silencing was found to be temperature dependent [32], RNA interference experiments were carried out at 30°C. P-element insertion lines, *Df(3 L)AC1*) and *slbo-Gal4 >UAS-GFP* were obtained from the Bloomington Stock Center, *slbo*^*1310*^*slbo-Gal4* from P. Rorth, *Df(2R)ED3* from R. Palmer and *UAS-TalinH-GFP* and *UAS-TalinH*^*R376A*^*-GFP* from N. Brown. RNA-interference transformants for *CalpB* (ID: 23037) and *CalpA* (ID: 35262) were purchased from VDRC, Austria, and for *if* (ID: 9623R-2), *mys* (ID: 1560R-1) and *rhea* (ID: 6831R-2) from NIG-FLY, Japan. We regret to note that the CalpA and CalpB deletion alleles, which were specifically generated during the course of our studies for the investigations of calpain functions, were unfortunately lost after the unexpected death of GÁ, and cannot be supplied to the scientific community.

### Nucleic acid isolation and amplification

Genomic DNA was isolated from single flies according to [33]. Total RNA was isolated with Trizol Reagent (Invitrogen) according to [34] and was treated with RNase-free DNase (Promega). RT-PCR was carried out as described in [35] with the CalpA_For, CalpA_Rev, and CalpB_For, CalpB_Rev oligonucleotide primer pairs. Primers 5CB1 and 3CB2 were used to PCR amplify the *CalpB* gene fragments from the deletion mutants. These fragments (approx. 1300 bp from *Calp*^*361*^ and 1150 bp from *CalpB*^*505*^) were isolated from agarose gels with the QIAGEN Gel Extraction Kit, and were sequenced subsequently in order to delineate the chromosomal breakpoints in the mutants. The breakpoints in the *CalpA*^*808*^ deletion mutant were determined in the same way, but with the 5HH and CP2 primers that produced a 7324 bp amplicon. The PCR analysis of the *CalpB*^*505*^*, UAS-TalinH-GFP* recombinant lines were performed with the 5CB1 and 3CB2 primers. Oligonucleotide primers and PCR conditions used in the present study are summarized in Additional file 4.

### DNA cloning and transformation

The *CalpB* genomic sequence was excised from the BAC RP98-7A5 vector (Roswell Park Cancer Institute Drosophila BAC Library) with *XbaI* (Fermentas). The approximately 6kbp fragment was isolated and inserted into the *Bluescript II KS* plasmid. Then the *CalpB* gene was released with *EcoRI* and *XhoI* (Fermentas) restriction enzymes and was ligated into the *pP{CaSpeR-4}* transforming vector. This construct (termed *CBG*^*26*^) that contains the entire ORF as well as 5’- and 3’-noncoding sequences (Figure 1D) was injected into the embryos according to standard procedures.

### Immunohistochemistry

Before dissection, females were fed for 24 hours with wet yeast paste at 25°C (at 30°C in RNA interference experiments), and ovaries were dissected in PBS buffer, fixed with 4% paraformaldehyde, washed 3x with PBS and 0.1% Triton-X100. Immunostainings were performed as described earlier [36]. Anti-β-PS integrin monoclonal antibody was obtained from DSHB and was diluted 1:10. As secondary antibodies, Alexa-488 and Alexa-546 conjugates (Invitrogen) were used in 1:500 dilutions. The actin cytoskeleton and the nuclei were stained with TRITC-Phalloidin (Sigma-Aldrich) and DAPI (Sigma-Aldrich), respectively. Examination and counting of the samples were conducted using an Olympus FV1000 LSM confocal laser scanning microscope. Images were obtained by combining multiple optical sections using the Olympus Fluoview Ver.1.7a Viewer software’s “intensity projection over z-axis” function. Figures were prepared with the Adobe Photoshop CS4 software.

### Western blotting

Western blot experiments were carried out according to [37]. Crude extracts (25 μg protein) representing different developmental stages of *Drosophila w*^*1118*^ strain were separated by SDS-PAGE [38]. Recombinant CalpB and CalpA were used as positive controls. Anti-CalpB and Anti-CalpA polyclonal antisera were generated against the full length recombinant proteins according to [15] and [39], respectively. Anti-Talin antibody was kindly provided by Nicholas Brown (Wellcome Trust/Cancer Research UK Institute, Department of Anatomy, University of Cambridge, Cambridge, UK). In embryo samples anti-β-Tubulin monoclonal antibody (Millipore/Upstate clone DM1A), while in other developmental stages anti-Actin polyclonal antibody (Sigma-Aldrich 20–33) were used to detect internal standards. Anti-CalpB and anti-Talin antibodies were used in 1:1000 dilution; anti-CalpA, anti-Actin and anti-Tubulin antibodies were applied in 1:2000 dilution. Anti-CalpB, anti-CalpA and anti-Actin antibodies were visualized with horseradish peroxidase conjugated anti-rabbit IgG raised in goat (Sigma-Aldrich A0545); the anti-Talin and anti-Tubulin antibodies were revealed with anti-mouse IgG secondary antibodies raised in rabbit (Sigma-Aldrich A9044). Secondary antibodies were diluted 1:10.000 in all of the experiments. A mixture of high and low molecular weight standards (Sigma-Aldrich), or Spectra Multicolor Broad Range and PageRuler (Fermentas) prestained protein ladder were used as molecular mass markers. Horseradish peroxidase activity was detected with Supersignal West Pico chemiluminescent reagent (Pierce) and the emitted light was captured by blue sensitive X-ray film (Agfa).

### Immunoprecipitation

20 adult flies of the *Drosophila w*^*1118*^ strain were ground and lysed in 200 μl lysis buffer (50 mM Tris, pH 7.5, 150 mM NaCl, 10 mM EDTA, 0.1% Triton X-100, 0.01% β-mercaptoethanol and 1xRoche Complete EDTA-free Protease Inhibitor Cocktail). Further steps of the immunoprecipitation were carried out according to [20] with the following modifications: the supernatants were immunoprecipitated with 40 μg of talin antibody adsorbed to Protein A Sepharose (Sigma-Aldrich). The immunocomplex was analyzed by Western blotting.

### In vitro cleavage of Drosophila talin with recombinant CalpB

100 mg of stage 2 *Drosophila* embryos were homogenized in 100 μl lysis buffer (50 mM Tris–HCl, pH 7.5, 150 mM NaCl, 2 mM dithiothreitol and 10 mM NaF) in ice. Cell debris were removed by centrifugation (2 min, 2000 g at 4°C) and the clear supernatant was treated with recombinant *Drosophila* CalpB that was prepared and assayed as described in [20]. The reaction was carried out in the absence and presence of 20 mM Ca^2+^ or 10 mM EDTA for 5 minutes at 25°C. The reaction was started by the addition of 1.5 μg CalpB enzyme and was terminated by the addition of SDS sample buffer containing 25 mM EDTA. Samples containing 3.2 μg proteins were boiled for 5 min in the presence of a pinch of dithioerytriol (Sigma-Aldrich) and were analyzed by Western blotting.

## Competing interests

The authors declare that they have no competing interests.

## Authors’ contributions

EK and FSP contributed to the work equally, and are considered as joint first authors of this article. FSP generated the calpain mutants and conducted their genetic analysis guided by GÁ. EK performed the molecular biology and biochemical assays under the supervision of VD. His work was continued by ÉK during the revision of the manuscript. MP did the RNAi experiments directed by PD. KS was involved in the analysis of border cell migration, and AC performed additional confocal microscopic analysis with GÁ. PF provided the theoretical background and initiated the study. GÁ acted as the principal investigator of the project, coordinated the research activities and drafted the first version of the manuscript. After his premature death VD took over the responsibilities of publication. All authors read and approved the final manuscript.

## Authors’ information

With great sadness we announce that our colleague GÁ died on 06.11. 2011. He drafted the first version of the manuscript, but his sudden departure prevented him from finishing his work. Although many of his experiments cannot be repeated any more due to the unfortunate loss of all fly stocks that he and his colleagues generated during the past few years, we would like to pay tribute to his memory with the publication of the results of his last efforts.

The present addresses of FSP and of KS are Zentrum für Molekulare Biologie der Universität Heidelberg, DKFZ-ZMBH Alliance, Heidelberg, Germany, and European Molecular Biology Laboratory, Meyerhofstraße 1, 69117 Heidelberg, Germany, respectively.

## Supplementary Material

Additional file 1 **Quantification of dorsal migration defects due to the*CalpB***^***505***^**mutation and to the downregulation of*mys, if*and*rhea*genes.** Colored bars represent the percentage of stage 10B egg chambers in which border cells displayed normal (yellow) and delayed (green) dorsal migration. Only the dorsal component of the movement was scored in all samples. The genotypes and the corresponding numbers of the egg chambers examined are indicated at the left and the right sides of the chart, respectively. Click here for file

Additional file 2 **Western blot analysis of CalpA expression in different developmental stages of*Drosophila.*** Extracts prepared from early (stage 2) and late (stage 17) embryos ( **A**), 1^st^ ,2^nd^ and 3^rd^ instar larvae, pupae, as well as from adult male and female flies (**B**), were separated by SDS-PAGE and probed with anti-CalpA antibody. 20 ng recombinant CalpA was loaded in the control lane. The positions of the molecular mass standards are denoted. Arrows indicate the main protein bands stained with the CalpA-specific antibody. The positions of internal standards α-tubulin in panel A and α-actin in panel B are also indicated by arrows. Click here for file

Additional file 3 **Temperature dependence of the*rhea*RNAi effect.** The diagram displays the quantification of border cell migration delays caused by the silencing of the *rhea* gene at 25°C and 30°C. Colored bars represent the proportion of stage 10A egg chambers in which border cells migrate 0% (pink), 1-25% (purple), 26-50% (dark blue), 51-75% (light blue), 75-99% (green) and 100% (yellow) of the wild-type distance. The genotypes and the corresponding numbers of the egg chambers examined are represented at the left and the right of the chart, respectively. Click here for file

Additional file 4 Oligonucleotide primers and conditions used for PCR amplifications. Click here for file
