# Supplementary figures and images for: CalpB modulates border cell migration in Drosophila egg chambers
Source: BMC Dev Biol. 2012 Jul 24;12:20. doi: 10.1186/1471-213X-12-20 (PMC3441222; doi:10.1186/1471-213X-12-20)

Additional file 1.

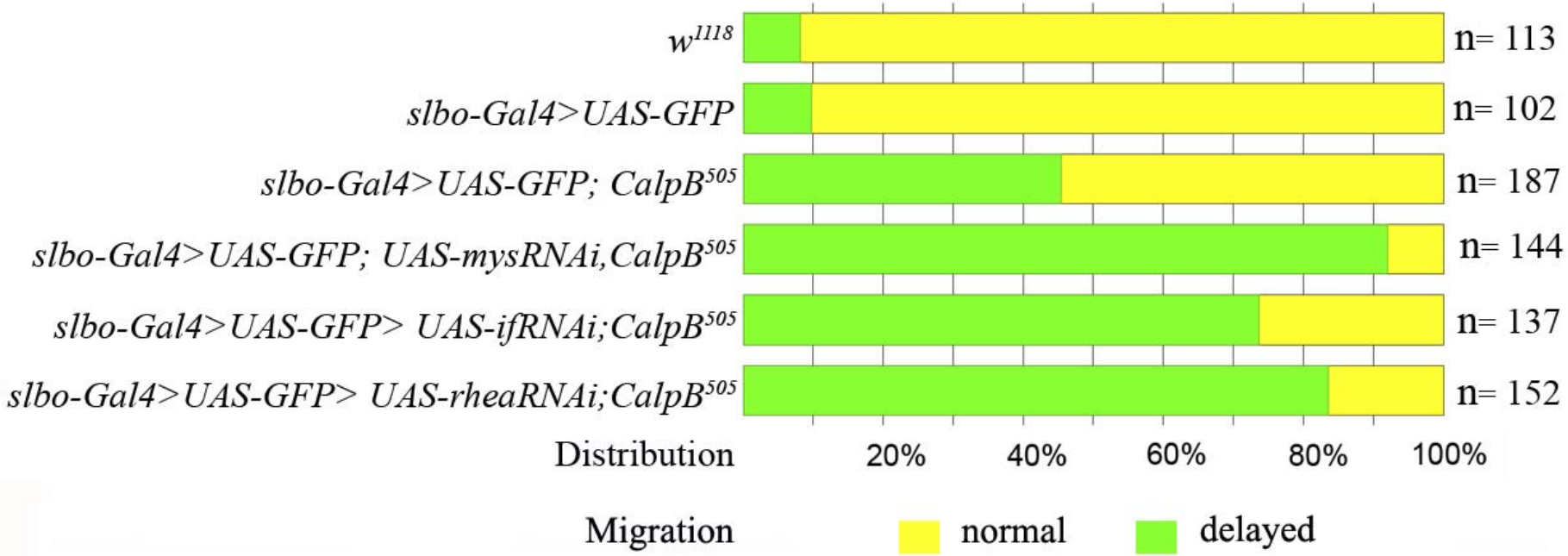

Supplement: Additional file 1 — Quantification of dorsal migration defects due to theCalpB505mutation and to the downregulation ofmys, ifandrheagenes. Colored bars represent the percentage of stage 10B egg chambers in which border cells displayed normal (yellow) and delayed (green) dorsal migration. Only the dorsal component of the movement was scored in all samples. The genotypes and the corresponding numbers of the egg chambers examined are indicated at the left and the right sides of the chart, respectively. [file 1471-213X-12-20-S1.pdf]

**A**

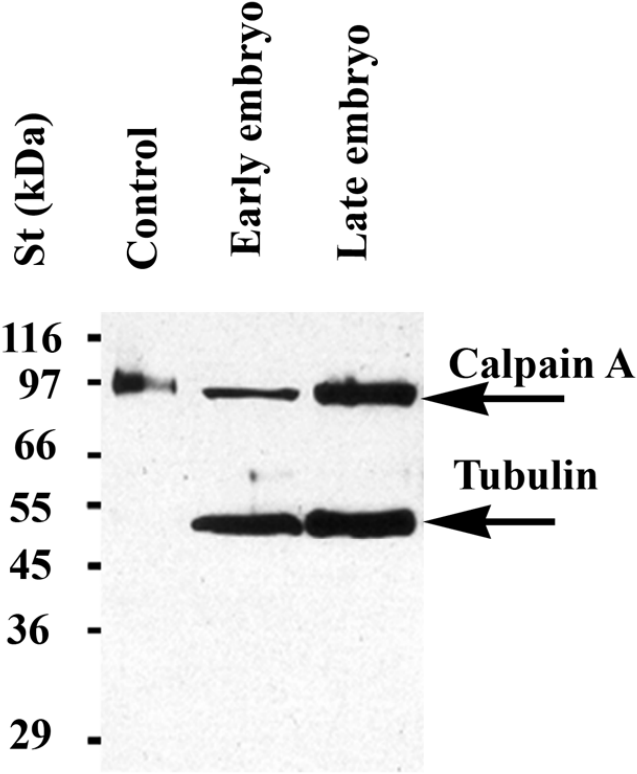

**B**

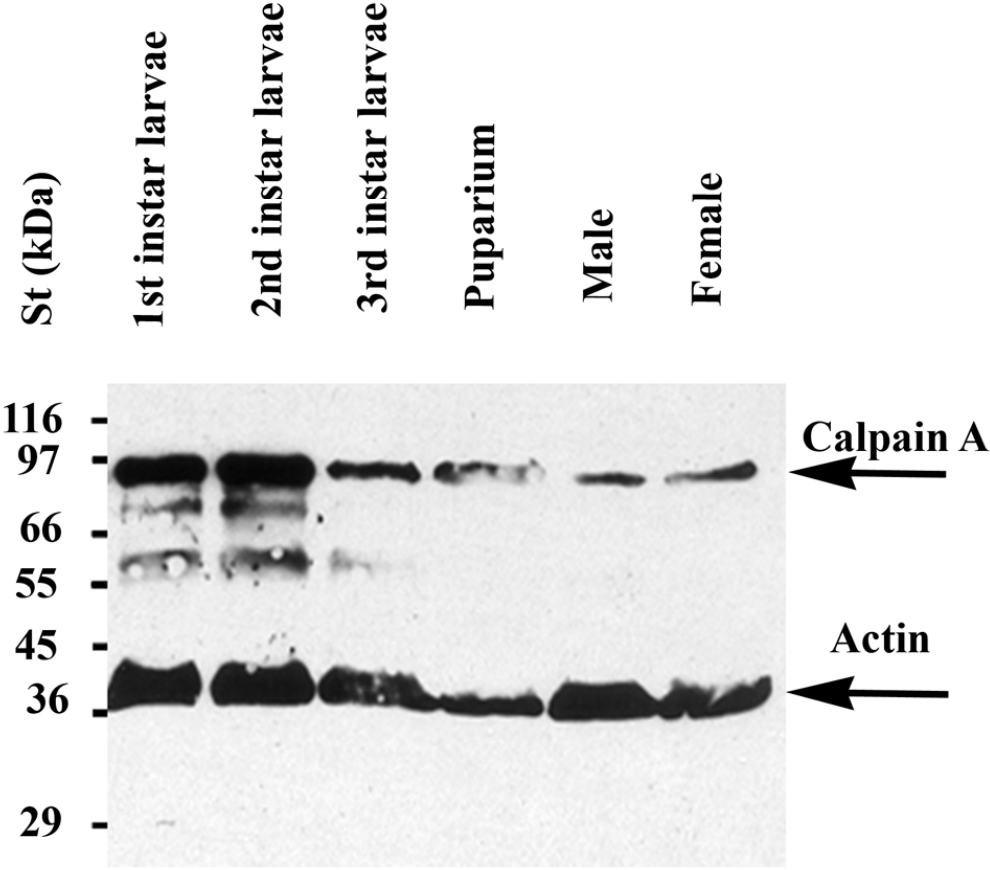

Supplement: Additional file 2 — Western blot analysis of CalpA expression in different developmental stages ofDrosophila. Extracts prepared from early (stage 2) and late (stage 17) embryos ( A), 1st ,2nd and 3rd instar larvae, pupae, as well as from adult male and female flies (B), were separated by SDS-PAGE and probed with anti-CalpA antibody. 20 ng recombinant CalpA was loaded in the control lane. The positions of the molecular mass standards are denoted. Arrows indicate the main protein bands stained with the CalpA-specific antibody. The positions of internal standards α-tubulin in panel A and α-actin in panel B are also indicated by arrows. [file 1471-213X-12-20-S2.pdf]

Additional file 3.

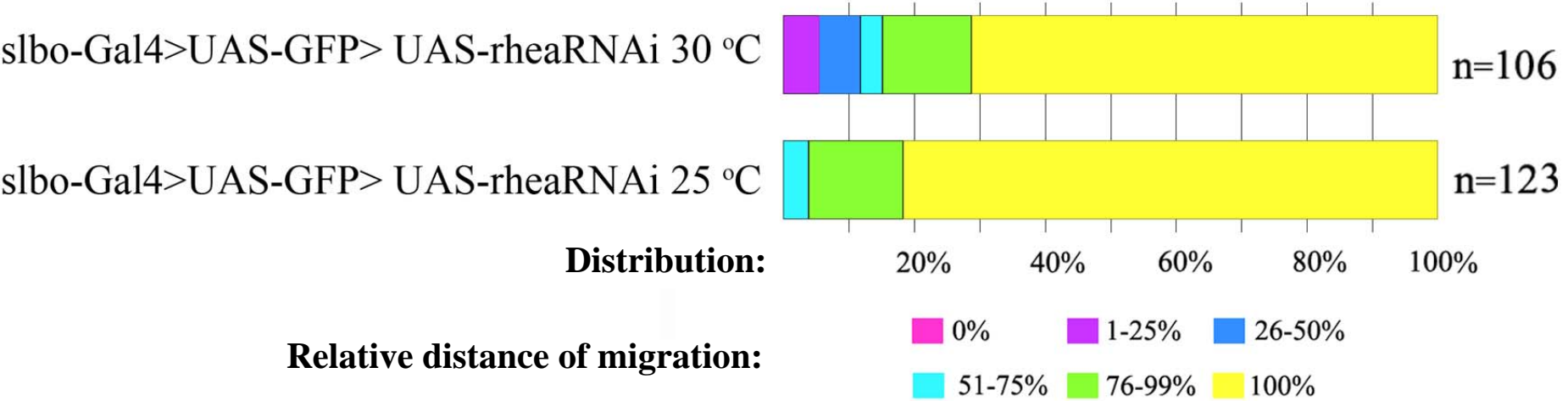

Supplement: Additional file 3 — Temperature dependence of therheaRNAi effect. The diagram displays the quantification of border cell migration delays caused by the silencing of the rhea gene at 25°C and 30°C. Colored bars represent the proportion of stage 10A egg chambers in which border cells migrate 0% (pink), 1-25% (purple), 26-50% (dark blue), 51-75% (light blue), 75-99% (green) and 100% (yellow) of the wild-type distance. The genotypes and the corresponding numbers of the egg chambers examined are represented at the left and the right of the chart, respectively. [file 1471-213X-12-20-S3.pdf]
