# Supplementary material for: CalpB modulates border cell migration in Drosophila egg chambers
Source: BMC Dev Biol. 2012 Jul 24;12:20. doi: 10.1186/1471-213X-12-20 (PMC3441222; doi:10.1186/1471-213X-12-20)
Supplement: Additional file 4 — Oligonucleotide primers and conditions used for PCR amplifications. [file 1471-213X-12-20-S4.pdf]

Target gene	Name	Sequence (5’-3’)	Annealing temperature (°C)	Extension time (s)
CalpA	CalpA_For	ACAATATTGAACAGCTGCCTG	56	120
	CalpA_Rev	TTCTGGTGAGCTATCACTCC		
	5HH	ACAGCATTGCTAATGCAGTTTAGC	58	180
	CP2	ACGCCGGGAAAACTGAAGAGACT		
CalpB	CalpB_For	TTTATACAGAACGCGCAAGG	58	60
	CalpB_Rev	CTTCCAGAACATGTGGATCC		
	5CB1	GGCACCTCGGGCACCTCAG	56	90-150
	3CB2	GCTCGGCGGCCTTCATCAT		
